# Effects of slide-board-based high-intensity interval versus moderate-intensity continuous training on aerobic and anaerobic capacity in young speed skaters

**DOI:** 10.1371/journal.pone.0343570

**Published:** 2026-02-27

**Authors:** Kai Zhang, Jing Qi, Peng Shi, Xin Xue, Yuanguo Liu

**Affiliations:** 1 School of Athletic Performance, Shanghai University of Sport, Shanghai, China; 2 School of Physical Education, Shandong University of Aeronautics, Binzhou, Shandong, China; 3 Department of Pathology, Affiliated Hospital of Binzhou Medical University, Binzhou, Shandong, China; 4 School of Physical Education, Shandong University of Technology, Zibo, Shandong, China; 5 School of Ice and Snow Sports, Ice and Snow Industry Research Institute, Shenyang Sport University, Shenyang, Liaoning, China; Erzurum Technical University: Erzurum Teknik Universitesi, TÜRKIYE

## Abstract

**Purpose:**

This study aimed to compare the effects of three slide-board training modalities—two high-intensity interval training protocols (HIIT1: 3 min work/2 min rest; HIIT2: 4 min work/1 min rest) and one moderate-intensity continuous training protocol (MICT: 20 min at 70% HRmax)—on aerobic and anaerobic capacities in young speed skaters.

**Methods:**

Twenty-seven youth speed skaters (15 males, 12 females) were randomly assigned to HIIT1, HIIT2, or MICT groups (n = 9 each). All participants completed a 4-week intervention (3 sessions/week) using a slide-board simulator. Aerobic capacity was assessed via maximal oxygen uptake (VO2max) and peak aerobic power output (Pmax) using an incremental cycle test. Anaerobic performance was evaluated with a 30-second Wingate test, including relative peak power (RPP), relative mean power (RMP), and fatigue index (FI). Pre- and post-test data were analyzed using two-way repeated measures ANOVA and paired t-tests.

**Results:**

Both HIIT1 and HIIT2 significantly improved VO2max, RPP, and RMP, and reduced FI (p < 0.05), whereas MICT showed no significant changes in any variable. HIIT2 demonstrated greater improvements in Pmax and anaerobic power metrics compared to HIIT1, though intergroup differences were not statistically significant. HIIT1 appeared to enhance fatigue resistance more effectively.

**Conclusion:**

Slide-board HIIT is an effective short-term training method for enhancing aerobic fitness and anaerobic power in youth speed skaters. HIIT2 (4 + 1 structure) may be more beneficial for sprinters requiring explosive power, while HIIT1 (3 + 2 structure) may suit middle- to long-distance skaters focusing on endurance and fatigue resistance. MICT alone appears insufficient to induce meaningful physiological adaptations in a 4-week period. These findings support the use of structure-specific HIIT protocols for sport-specific conditioning in speed skating.

## Introduction

Speed skating requires the concurrent development of two contrasting yet complementary energy systems: aerobic capacity for sustained pacing and anaerobic power for decisive bursts during starts, accelerations, and sprints [1]. This dual demand creates unique challenges for athletes and coaches, who must adopt training strategies that simultaneously enhance both endurance and explosive output. Recent speed skating–specific work has emphasized that performance in short- and middle-distance events depends not only on global physiological capacity but also on phase-specific acceleration and start performance, as reflected by reliable split-time and velocity profiles over the first 100 m of racing [2]. In parallel, strength and conditioning guidelines for competitive speed skaters highlight the need for integrated development of strength, power, and energy system capacity across race phases, as well as careful management of injury risk in short- to middle-distance athletes [3].

Maximal oxygen uptake (VO2max) is widely recognized as a cornerstone of endurance capacity. Elite speed skaters typically reach > 60 mL/kg/min for males and > 50 mL/kg/min for females [4], levels higher than those of team-sport athletes [5] yet still 12–18% below specialized endurance athletes such as cross-country skiers [6]. Two main factors contribute to this limitation: reduced femoral blood flow of up to 30% caused by the crouched skating posture [7], and methodological discrepancies, since VO2max values measured on-ice are typically 15–20% lower than treadmill-based tests [8]. Data from Chinese national teams confirm a 7–12% VO2max deficit relative to international peers [9], although targeted interventions can produce improvements of 4.3–6.1% [10].

Anaerobic power is equally critical, particularly for explosive efforts decisive to performance. World-class male skaters produce 16.6–24.4 W/kg peak and 11.2–14.2 W/kg mean power, while females achieve 11.4–20.0 W/kg peak and 9.3–12.6 W/kg mean values. Each 1 W/kg increase in peak power correlates with a 0.82-second improvement over 500 m, while each 1 W/kg increase in mean power corresponds to time reductions of nearly one second in the 1500 m [11,12]. However, Chinese skaters have been reported to show 18–22% lower anaerobic power than global elites, highlighting a performance gap requiring targeted intervention.

High-intensity interval training (HIIT) has been widely recognized as a time-efficient method to enhance both aerobic and anaerobic systems, eliciting two- to three-fold greater per-session VO2max improvements compared to moderate-intensity continuous training (MICT), with substantially lower training volume [13]. Beyond athletic performance, HIIT has also been increasingly applied in health and rehabilitation settings, suggesting benefits that extend to broader populations [14]. Consistent with its time-efficient nature, HIIT can elicit metabolic adaptations (e.g., improvements in lipid and glucose metabolism) that are thought to be underpinned, at least in part, by cellular signaling responses and mitochondrial adaptations [15]. Mechanistically, HIIT activates the AMPK–PGC-1α pathway, stimulating mitochondrial biogenesis and oxidative enzyme activity [16], while simultaneously improving neuromuscular specificity and power output in movements similar to sport performance [17]. Indeed, 6 weeks of HIIT has been shown to improve VO2max by 6–12% in endurance athletes, comparable to adaptations from 12 weeks of MICT [18]. MICT nevertheless retains a central role, especially during transition periods, by increasing capillary density and oxidative enzyme capacity [19], optimizing fat oxidation [20], and preserving performance even under reduced training loads [21]. Despite extensive evidence demonstrating the distinct effects of HIIT and MICT, these findings have been derived primarily from treadmill and cycle ergometer studies, leaving it unclear whether they apply under slide-board conditions that replicate skating-specific demands.

The slide board provides a land-based modality closely replicating the biomechanics of skating, including low crouched posture, lateral propulsion, and sustained hip and knee flexion. Compared with treadmill or cycle ergometry, slide-board testing better reflects skating-specific physiological demands [22,23]. Recent validation studies confirmed the reliability of incremental slide-board tests for both VO2max and anaerobic assessments, showing closer alignment with on-ice capacity than traditional ergometry [24,25]. Beyond testing, slide-board interval training has also been shown to improve both aerobic capacity and sport-specific skating fitness, and may even be superior to cycle-ergometer–based training in ice hockey players [26]. Nevertheless, most research has focused on methodological validation, with little evidence addressing training adaptations under slide-board conditions.

Moreover, no studies to date have systematically compared the effects of distinct long-interval HIIT structures in speed skating. The design of work-to-rest ratios is particularly relevant, as it determines the balance between aerobic strain and anaerobic stress. For example, a 3-min work/2-min recovery format (HIIT1) may allow longer time accumulated at high oxygen uptake, favoring aerobic development, whereas a 4-min work/1-min recovery format (HIIT2) may induce greater glycolytic stress and lactate accumulation, potentially benefiting anaerobic performance. While these mechanisms are theoretically plausible, empirical verification remains lacking.

Therefore, the present study aimed to compare the effects of HIIT and MICT, implemented using slide-board training, on aerobic and anaerobic capacity in young speed skaters. A secondary aim was to examine whether two long-interval HIIT formats (HIIT1: 3/2; HIIT2: 4/1) would induce differential adaptations. We hypothesized that: (i) HIIT would elicit greater improvements than MICT; (ii) HIIT1 would preferentially enhance aerobic capacity; and (iii) HIIT2 would be more effective for anaerobic performance.

## Methods

### Participants

Initially, 30 healthy young speed skaters were recruited from Shenyang Sport University. Participants were recruited voluntarily through internal announcements and academic course platforms. Recruitment occurred from 24 May 2024–24 June 2024. All subjects held a national second-level athlete certificate or above and had at least 5 years of systematic training experience. None were smokers, and all were free of any current injuries or medical conditions that could affect the study outcomes.

During the subsequent screening and intervention phases, three participants withdrew due to personal reasons or poor compliance (one from each training group). Therefore, 27 participants (15 males and 12 females) completed the full intervention and were included in the final analysis.

An a priori sample size estimation was performed using G*Power (v3.1.9) for a two-factor repeated-measures ANOVA (group × time). Assuming a medium effect size (f = 0.38) [27], α = 0.05, and power (1-β) = 0.80. The minimum required total sample size was 21 participants. To account for potential invalid data or attrition, we inflated the target sample size by 20%, yielding an expected minimum sample size of 27 participants. Therefore, 30 participants were recruited at baseline.

Inclusion criteria were as follows: (1) age between 18 and 22 years; (2) certified speed skaters at or above the national second-class athlete level; (3) body mass index (BMI) < 25 kg/m². In cases of slightly elevated BMI due to high muscle mass, eligibility was determined based on a combined assessment of body fat percentage and metabolic indicators; (4) resting blood pressure below 130/85 mmHg. For those with a blood pressure range of 130–139/85–89 mmHg, clinical evaluation was required to confirm the safety of participating in moderate-to-high intensity training; (5) no history of cardiovascular disease, thrombosis, neuromuscular disorders, hematologic abnormalities, musculoskeletal injuries, or other chronic illnesses; (6) not taking any medications or supplements that could influence the study results.

Exclusion criteria included: (1) individuals deemed high-risk or unsuitable for aerobic exercise based on the Physical Activity Readiness Questionnaire (PAR-Q) and the International Physical Activity Questionnaire (IPAQ); (2) recent musculoskeletal injury or surgery within the past three months; (3) history of severe cardiovascular, respiratory, or musculoskeletal diseases; (4) refusal to provide written informed consent or inability to comply with study procedures.

All participants were adults (≥ 18 years). Written informed consent was obtained from every participant before any study procedures; no minors were enrolled, and no consent waiver was sought. Consent forms were retained according to institutional policy. The study was approved by the Ethics Committee of Shanghai University of Sport (Approval No. 10272024RT024; approved on: 22 May 2024) and conducted in accordance with international ethical standards (including the Declaration of Helsinki). Personally identifiable information was not included in the analytical dataset; data were de-identified using study IDs.

### Study design

This study employed a 4-week parallel-group randomized controlled design to compare the effects of different slide board training protocols on aerobic and anaerobic capacities in speed skaters. A total of 30 eligible participants were recruited and randomly assigned to one of three groups (n = 10 per group) using a random number table. Each group completed the following training protocols three times per week:

HIIT1 group: 3 minutes of sliding followed by 2 minutes of rest, repeated for 4 sets; training intensity was maintained at 85% of maximal heart rate (HRmax) [28];

HIIT2 group: 4 minutes of sliding followed by 1 minute of rest, repeated for 4 sets; intensity matched that of the HIIT1 group [29];

MICT group: 20 minutes of continuous sliding at 70% HRmax [30].

To develop HIIT protocols that align with the specific demands of speed skating, the training models proposed by Laursen et al. [29] were referenced and further refined based on expert consultation. As a result, two representative long-interval HIIT structures were selected for implementation in this study. The rationale for selecting two distinct long-interval HIIT formats (HIIT1: 3/2 and HIIT2: 4/1) was to examine how different work-to-rest ratios modulate the balance between aerobic strain and anaerobic stress, thereby allowing direct comparison of their adaptation effects in young speed skaters. Specifically, HIIT1 was expected to favor greater accumulated time at high oxygen uptake due to longer recovery, whereas HIIT2 was expected to impose higher glycolytic stress and lactate accumulation owing to shorter recovery. All training sessions lasted 20 min in each group. However, because the work–rest structures differed (HIIT1: 4 × [3-min work + 2-min rest]; HIIT2: 4 × [4-min work + 1-min rest]; MICT: 20 min continuous), the total work duration per session was 12, 16, and 20 min, respectively. Thus, although intensity was prescribed using heart-rate targets (HIIT: 85% HRmax; MICT: 70% HRmax), total workload was not fully equated across groups.

All training was performed on a commercially available skating-specific slide board (UltraSlide 10, UltraSlide, Lake Bluff, IL, USA). The board consisted of a low-friction synthetic sliding surface mounted on a premium plywood base with fixed hardwood bumpers at both ends. For all participants, the effective sliding length was set to 2.1 m (7 ft), and the usable width of the surface was 0.60 m (23.5 in). The board was placed on a level indoor floor in the same facility as the testing sessions. To standardize the interface with the sliding surface, all participants wore identical slide-board overshoes over their training shoes. This setup was chosen to reproduce the lateral push-off and gliding motion patterns of speed skating while ensuring consistent mechanical characteristics across participants.

Slide-board skating was selected as a skating-specific dryland modality that reproduces key movement features relevant to speed skating (e.g., repeated lateral push-offs) [25]. Evidence from skating-specific assessments supports the physiological validity of slide-board skating: incremental slide-board tests demonstrate criterion validity versus treadmill skating and correlate with skating performance indices [24], and slide-board skating elicits similar VO2 kinetics and muscle deoxygenation kinetics compared with treadmill skating at matched relative intensity [30]. During each training session, participants performed slide-board exercise with a cadence cue to standardize movement rhythm [31]. Heart rate was continuously monitored, and workload/cadence was adjusted to ensure that the average heart rate within each work interval met the prescribed target intensity.

Training intensity was individualized based on each participant’s HRmax, which was determined through a graded exercise test conducted during the pre-experimental phase. Heart rate was monitored continuously using Polar heart rate monitors, and workload/cadence was adjusted as needed to keep participants within the prescribed target zones (HIIT: 85% HRmax; MICT: 70% HRmax). All sessions were supervised by experienced performance coaches. Participants were scheduled to complete 12 supervised training sessions over 4 weeks (3 sessions per week), and attendance was recorded at each session. They were instructed to maintain their usual sleep and dietary routines and to keep daily logs of sleep duration, dietary intake, and any additional vigorous physical activity outside the intervention. These logs were reviewed weekly to identify major deviations and to help control potential confounding variables and ensure training specificity. All training sessions were performed indoors in the same facility as the testing sessions, under stable laboratory conditions comparable to those used for testing. All training and testing sessions were supervised by experienced performance coaches and sports medicine staff. Prior to participation, all athletes underwent a health screening to exclude cardiovascular, neuromuscular, or other high-risk conditions. During training and testing, heart rate and subjective symptoms (e.g., RPE, dizziness, chest discomfort) were monitored in real time, and any participant showing abnormal signs would have the session immediately stopped and be evaluated by the medical staff. Before inclusion, participants completed the PAR-Q to assess potential health risks [32], ensuring their safety for participation. Additionally, the IPAQ was used to assess baseline and within-study physical activity levels [33], providing a reference for interpreting training outcomes.

Both pre- and post-intervention assessments were scheduled within 7 days prior to the intervention and exactly 72 hours after the final training session. The testing sequence was standardized across all participants: aerobic capacity tests were conducted first, followed by anaerobic capacity tests, to avoid fatigue-induced interference with aerobic performance. All tests were conducted between 09:00 and 11:30 a.m., and efforts were made to ensure each participant was tested at the same time of day for both pre- and post-tests. Assessments were performed in the same climate-controlled laboratory, with the ambient temperature maintained at 22.5 ± 1°C. All tests were administered by the same team of trained technicians using identical equipment and standardized operating procedures. Participants were instructed to refrain from vigorous exercise, alcohol consumption, and the use of metabolism-affecting medications within 48 hours before testing. On the test day, participants were required to fast for at least 8 hours, though water intake was permitted. Prior to testing, all subjects performed a 5–10 minute low-intensity warm-up cycling session (60 W) to ensure both safety and accuracy during the tests.

A dedicated team was assigned to data entry and quality control. A double-entry and cross-validation system was implemented to ensure data accuracy. During statistical analysis, group allocation was blinded to the analysts in order to minimize subjective bias and ensure the objectivity and scientific rigor of the study outcomes.

### Incremental exercise test

To assess aerobic capacity, a graded exercise test (GXT) was conducted using a cycle ergometer (Ergoselect 100K, Bitz, Germany). This ergometer was used exclusively for the GXT at both pre- and post-intervention assessments, and its braking system and cadence display were checked according to the manufacturer’s calibration procedures before each testing day. Gas exchange was continuously monitored using a cardiopulmonary exercise testing system (MAX II, Physio-Dyne Instrument Corp., USA) with breath-by-breath analysis [34,35]. Prior to testing, the system was calibrated following the manufacturer’s instructions, including volume calibration with a 3-liter syringe and gas calibration for accurate composition readings. The test protocol began at an initial workload of 50 W, with increments of 25 W every 2 minutes. Cadence was maintained at 60–70 rpm until volitional exhaustion was reached [36]. Heart rate was recorded in real time using a Polar H10 heart rate monitor (Polar Electro Oy, Finland). VO2max was defined as the highest 15-second average oxygen uptake recorded during the test. The attainment of VO2max was confirmed if at least two of the following four criteria were met: (1) a plateau in VO2 despite increasing workload; (2) respiratory exchange ratio (RER) ≥ 1.15; (3) rating of perceived exertion (RPE, Borg 6–20 scale) ≥ 17; (4) measured HR ≥ 90% of age-predicted HRmax (HRmax = 220 – age). The test was immediately terminated if participants exhibited signs of fatigue, plateaued VO2 or heart rate, or reported discomfort such as dizziness, nausea, or chest tightness. Onsite professionals evaluated whether the participant could safely continue. Maximal aerobic power output (Pmax) was estimated using the following formula:

Pmax (W) = Power at last completed stage (W) + [t (s)/ stage duration (s)] × workload increment (W), where t represents the time elapsed in the uncompleted final stage [37]. Although no electrocardiogram was used during the test, qualified coaches and medical staff were present to monitor participant safety based on real-time HR data and subjective feedback (e.g., RPE and physical symptoms). Testing was immediately stopped in the event of excessive fatigue or abnormal symptoms to ensure participant safety.

### Wingate anaerobic test

The Wingate Anaerobic Test was conducted using a cycle ergometer (Cyclus2, RBM elektronik-automation GmbH, Leipzig, Germany) to evaluate participants’ anaerobic power output capacity [38]. This ergometer was used exclusively for the Wingate protocol at both pre- and post-intervention time points and was warmed up and calibrated in accordance with the manufacturer’s instructions before each testing day. This test is a widely accepted method for assessing short-term maximal power and muscle fatigue resistance. Prior to testing, participants performed a 5-minute low-intensity warm-up at a workload of 1.0 W/kg, maintaining a cadence of 80 rpm to enhance neuromuscular activation. Following a 2-minute rest period, the formal Wingate test was initiated. The resistance was set at 7.5% of the participant’s body mass (i.e., 0.075 kg × body weight in kg) [39]. Upon the verbal cue “start,” resistance was applied instantaneously, and participants were instructed to pedal at maximal cadence for 30 seconds. Seat height was individually adjusted according to participant stature to maintain approximately 25–35°of knee flexion at the bottom of the pedal stroke. All participants used identical cycling equipment, shoes, and seat settings to minimize variability due to test conditions. Standardized verbal encouragement was provided throughout the test, and participants were instructed to keep their hips in contact with the saddle to ensure consistent effort and posture. During the test, the Cyclus2 display showing instantaneous power and cadence was visible to all participants, who were encouraged to use this information, together with standardized verbal encouragement, to maintain maximal effort throughout the 30-s bout. Visual feedback conditions were identical for all participants across pre- and post-tests to ensure consistent testing conditions. During the test, power output was recorded every 5 seconds to generate a power–time curve. Based on this curve, the following parameters were calculated: (1) Relative Peak Power (RPP), defined as the highest 5-second power output divided by body mass (W/kg); (2) Relative Mean Power (RMP), defined as the 30-second average power output per kilogram of body mass (W/kg); (3) Fatigue Index (FI), the traditional Wingate fatigue index is commonly expressed as a percentage: FI% = [(PP − Pmin)/ PP] × 100 [40], where PP is peak power and Pmin is the minimum power recorded during the 30-s Wingate test. In the present study, we report the Cyclus2-derived fatigue index (FI, W/s), a facility-specific (manufacturer-defined) metric provided by the ergometer software. This FI reflects the rate of power decline (i.e., fatigue development) from PP to Pmin [41] and is calculated as: FI (W/s) = (PP − Pmin)/ (tmin − tPP), where tPP is the time point at which PP occurs and tmin is the time point at which Pmin is recorded [42]. Although FI (W/s) differs from the conventional percentage-based FI% metric, using the device’s built-in algorithm ensured consistent measurement across all participants. All tests were supervised and conducted by the same trained researcher to ensure procedural consistency and data reliability.

### Statistical analyses

All statistical analyses were performed using IBM SPSS Statistics version 26.0 (IBM Corp., Armonk, NY, USA). Continuous variables that met the assumption of normal distribution were presented as mean ± standard deviation (Mean ± SD). The Shapiro–Wilk test and Levene’s test were used to assess normality and homogeneity of variances, respectively. For normally distributed variables with equal variances, a 2 (time: pre, post) × 3 (group: HIIT1, HIIT2, MICT) mixed-design repeated-measures ANOVA (2 × 3 RM ANOVA) was performed to examine the main effects of time, group, and their interaction. When a significant interaction effect was observed, simple effect analyses were conducted using paired-sample t-tests (within-group comparisons) and independent-sample t-tests (between-group comparisons). Effect sizes (ES) were calculated as partial eta squared (η²) for ANOVA main and interaction effects and Cohen’s dz for paired-sample t-tests and Cohen’s d for independent-sample t-tests. Partial η² values of 0.01, 0.06, and 0.14 were interpreted as small, medium, and large effects, respectively, and Cohen’s d(z) values of 0–0.19, 0.20–0.49, 0.50–0.79, and ≥0.80 were interpreted as trivial, small, moderate, and large, respectively [43].

## Results

### General characteristics

A total of 30 eligible participants were recruited for this study and randomly assigned to the HIIT1, HIIT2, and MICT groups. During the intervention, three participants withdrew due to personal reasons or poor compliance (one from each group). Ultimately, 27 participants completed the full 4-week, 12-session training and testing protocols and were included in the final analysis (n = 9 per group). Among these 27 participants, no training session or test was terminated early for safety reasons, and no adverse events were reported.

Table 1 presents the baseline characteristics of the three groups, including age, height, body weight, body mass index (BMI). Shapiro–Wilk tests for normality and Levene’s tests for homogeneity of variances indicated that all variables were normally distributed and met the assumption of homogeneity (all p > 0.05). Therefore, one-way analysis of variance (ANOVA) was used for group comparisons. The results showed no significant differences among the three groups in age (F(2, 24) = 1.091, p = 0.352), height (F(2, 24) = 0.618, p = 0.547), weight (F(2, 24) = 0.681, p = 0.516), or BMI (F(2, 24) = 1.566, p = 0.230), indicating good baseline comparability in demographic and training-related characteristics across groups(Table 1).

**Table 1:** Baseline characteristics of participants in the HIIT1, HIIT2, and MICT groups.

Variables	HIIT1 (n = 9)	HIIT2 (n = 9)	MICT (n = 9)	F	p-Values
Age, years	21.00 ± 1.50	21.11 ± 1.36	20.22 ± 1.30	1.091	0.352
Height, cm	174.67 ± 8.86	178.11 ± 10.45	173.44 ± 8.26	0.618	0.547
Weight, kg	65.38 ± 13.45	72.04 ± 13.86	68.91 ± 8.26	0.681	0.516
BMI, kg/m²	21.21 ± 2.57	22.49 ± 2.11	22.84 ± 1.29	1.566	0.230

Data are presented as mean ± SD. BMI: body mass index, HIIT: high-intensity interval training, MICT: moderate-intensity continuous training.

### Cardiorespiratory fitness

Prior to the intervention, Shapiro–Wilk tests indicated normal distribution for VO2max and Pmax (all p > 0.05). Levene’s tests showed no significant baseline differences across groups in VO2max (F(2,24) = 1.385, p = 0.270) and Pmax (F(2,24) = 1.754, p = 0.195).

For VO2max, a significant main effect of time was observed (F(1,24) = 14.215, p = 0.001, η^2^ = 0.372), whereas the group effect was not significant (F(2,24) = 0.090, p = 0.914, η^2^ = 0.007). The time × group interaction was also not significant (F(2,24) = 1.921, p = 0.168, η^2^ = 0.138). Paired-sample t-tests showed significant improvements in VO2max for HIIT1 (t = −2.456, p = 0.040, dz = 0.82) and HIIT2 (t = −2.673, p = 0.028, dz = 0.89), but not for MICT (t = −1.306, p = 0.228, dz = 0.44) (Table 2).

**Table 2:** Aerobic capacity variables measured before and after intervention.

Variable	Group	Pre-test	Post-test	t	p	Cohen’s dz
VO2max (mL/kg·min)	HIIT1(n = 9)	43.37 ± 3.50	43.89 ± 3.61*	−2.456 (8)	0.040	0.82
	HIIT2(n = 9)	42.70 ± 5.02	43.68 ± 5.71*	−2.673 (8)	0.028	0.89
	MICT(n = 9)	42.58 ± 4.84	42.82 ± 4.84	−1.306 (8)	0.228	0.44
Pmax(W)	HIIT1(n = 9)	217.17 ± 20.22	220.00 ± 21.32	−1.963 (8)	0.085	0.65
	HIIT2(n = 9)	213.13 ± 28.77	218.40± 27.45*	−3.247 (8)	0.012	1.08
	MICT(n = 9)	211.76 ± 24.77	213.33 ± 25.63	−1.153 (8)	0.282	0.38

Data are presented as mean ± SD. VO2max: maximal oxygen uptake, Pmax: maximal aerobic power output, HIIT: high-intensity interval training, MICT: moderate-intensity continuous training, *p < 0.05: indicates a significant difference compared with the pre-test.

For Pmax, there was a significant time effect (F(1,24) = 14.242, p =0.001, η^2^ = 0.372), but no significant main effect of group (F(2,24) = 0.134, p = 0.875, η^2^ = 0.011) or interaction effect (F(2,24) = 1.620, p = 0.219, η^2^ = 0.119). Paired-sample t-tests revealed a significant improvement in HIIT2 (t = −3.247, p = 0.012, dz = 1.08), while HIIT1 showed a trend (t = −1.963, p = 0.085, dz = 0.65) and MICT did not change significantly (t = −1.153, p = 0.282, dz = 0.38) (Table 2).

### Wingate anaerobic test: Power metrics and fatigue index

Prior to the intervention, Shapiro–Wilk tests indicated normal distribution for RPP, RMP, and FI (all p > 0.05). Levene’s tests showed no significant baseline differences across groups in RPP (F(2,24) = 0.745, p = 0.485), RMP (F(2,24) = 1.587, p = 0.225), or FI (F(2,24) = 0.059, p = 0.943).

For RPP, ANOVA revealed a significant main effect of time (F(1,24) = 12.340, p = 0.002, η^2^ = 0.340), a non-significant group effect (F(2,24) = 2.202, p = 0.132, η^2^ = 0.155), and a significant time × group interaction (F(2,24) = 3.535, p = 0.045, η^2^ = 0.228). HIIT1 (t = −2.635, p = 0.030, dz = 0.88) and HIIT2 (t = −2.857, p = 0.021, dz = 0.95) improved significantly, whereas MICT did not (t = 0.212, p = 0.838, dz = 0.07) (Table 3).

**Table 3:** Anaerobic capacity variables measured before and after intervention.

Variable	Group	Pre-test	Post-test	t	p	Cohen’s dz
Relative Peak Power (W/kg)	HIIT1 (n = 9)	9.67 ± 1.38	11.63 ± 1.80*#	−2.635 (8)	0.030	0.88
	HIIT2 (n = 9)	9.74 ± 1.07	11.49 ± 2.12*&	−2.857 (8)	0.021	0.95
	MICT (n = 9)	9.67 ± 1.03	9.59 ± 1.11	0.212 (8)	0.838	0.07
Relative Mean Power (W/kg)	HIIT1 (n = 9)	7.34 ± 1.23	8.77 ± 1.96*#	−2.636 (8)	0.030	0.88
	HIIT2 (n = 9)	6.99 ± 0.68	7.94 ± 1.06*&	−2.881 (8)	0.020	0.96
	MICT (n = 9)	6.83 ± 1.01	6.88 ± 0.83	−0.282 (8)	0.785	0.09
Fatigue Index (W/s)	HIIT1 (n = 9)	13.53 ± 4.29	12.18 ± 3.88*	2.352 (8)	0.047	0.78
	HIIT2 (n = 9)	13.39 ± 4.94	12.00 ± 4.25*	2.915 (8)	0.019	0.97
	MICT (n = 9)	14.54 ± 4.16	14.52 ± 4.42	0.070 (8)	0.946	0.02

Data are presented as mean ± SD. RPP: relative peak power, RMP: relative mean power, FI: fatigue index, HIIT: high-intensity interval training, MICT: moderate-intensity continuous training, *p < 0.05: indicates a significant difference compared with the pre-test, #p < 0.05: indicates a significant difference in the post-test between the HIIT1 and MICT groups, &p < 0.05: indicates a significant difference in the post-test between the HIIT2 and MICT groups.

For RMP, there was a significant main effect of time (F(1,24) = 13.778, p = 0.001, η^2^ = 0.365), a non-significant group effect (F(2,24) = 2.881, p = 0.076, η^2^ = 0.194), and a significant interaction (F(2,24) = 3.459, p = 0.048, η^2^ = 0.224). Both HIIT1 (t = −2.636, p = 0.030, dz = 0.88) and HIIT2 (t = −2.881, p = 0.020, dz = 0.96) showed significant gains, while MICT did not (t = −0.282, p = 0.785, dz = 0.09) (Table 3).

For FI, a significant time effect was observed (F(1,24) = 11.596, p = 0.002, η^2^ = 0.326), while the group effect (F(2,24) = 0.510, p = 0.607, η^2^ = 0.041) and interaction (F(2,24) = 2.672, p = 0.083, η^2^ = 0.187) were not significant. Paired-sample t-tests showed significant FI reductions in HIIT1 (t = 2.352, p = 0.047, dz = 0.78) and HIIT2 (t = 2.915, p = 0.019, dz = 0.97), but not in MICT (t = 0.070, p = 0.946, dz = 0.02) (Table 3).

## Discussion

The results demonstrated that both HIIT protocols outperformed MICT in improving aerobic and anaerobic parameters, suggesting that structured and optimized slide board-based HIIT provides superior benefits in enhancing both cardiorespiratory endurance and short-duration high-intensity performance, with strong sport-specific adaptability for speed skating.

VO2max, a core indicator of cardiovascular endurance and aerobic metabolic potential, reflects the entire physiological pathway from pulmonary oxygen uptake to muscular oxygen utilization [44]. HIIT, through repeated bouts of short-duration high-intensity exercise, can effectively improve cardiac output and peripheral oxygen extraction, while promoting mitochondrial biogenesis and increased capillary density [45]. In this study, both HIIT1 and HIIT2 were conducted at 85% HRmax with moderate-to-long recovery intervals, which may have elicited substantial central and peripheral adaptations, resulting in significant improvements in VO2max. In contrast, the MICT protocol—performed at 70% HRmax—may not have provided sufficient physiological stress to induce notable adaptations. These findings are consistent with previous studies by Wang Z et al. [46] and Lasso-Quilindo et al. [41], which demonstrated significant improvements in VO2max following short-term HIIT interventions.

Regarding Pmax, only the HIIT2 group showed a statistically significant post-intervention improvement (p = 0.012), while HIIT1 demonstrated a non-significant upward trend (p = 0.085), and the MICT group showed virtually no change. Pmax represents the upper limit of aerobic power output, regulated by a combination of oxygen uptake, energy conversion efficiency, and neuromuscular coordination [47,48]. The HIIT2 protocol, featuring a 4-minute high-intensity bout followed by a 1-minute recovery interval (“4+1” structure), provides a greater integrative stimulus for both glycolytic and oxidative systems per unit time. This may enhance the recruitment of type II muscle fibers and improve neuromuscular coordination, thereby contributing to the observed Pmax increase [49]. Furthermore, the shorter recovery periods in the “4+1” structure may prevent complete physiological recovery between intervals, promoting greater cumulative metabolic and neuromuscular adaptations.

The selection of the two long-interval HIIT structures (HIIT1: 3/2; HIIT2: 4/1) was based on the hypothesis that different work-to-rest ratios would differentially modulate aerobic and anaerobic demands. Specifically, HIIT1, with longer recovery, was expected to allow greater accumulated time at high oxygen uptake, favoring aerobic adaptations, whereas HIIT2, with shorter recovery, was anticipated to impose greater glycolytic stress and lactate accumulation, favoring anaerobic development. Our findings partially support this rationale: HIIT2 significantly improved Pmax, while HIIT1 showed only a non-significant trend. However, given the absence of significant interaction effects and the small sample size, these interpretations should be considered preliminary. Further studies with larger cohorts and longer interventions are warranted to clarify whether distinct long-interval HIIT formats confer event-specific advantages in speed skating.

Improvements in both RPP and RMP reflect enhanced short-duration high-intensity exercise capacity [50], which is primarily influenced by the function of the phosphagen system [51] and the fast glycolytic system [52]. Due to its high intensity and short recovery intervals, HIIT can significantly improve the mobilization efficiency of the ATP-PCr system [53], enhance recruitment of type II muscle fibers, and increase glycolytic enzyme activity, thereby boosting explosive power output [54]. Although the magnitude of improvement in power-related metrics was comparable between HIIT1 and HIIT2, the effect size was slightly greater for HIIT2 (dz = 0.95 vs. 0.88), suggesting that shorter recovery intervals may provide stronger neuromuscular stimulation and greater accumulation of metabolic stress. Moreover, between-group t-tests revealed that both HIIT groups exhibited significantly higher RPP and RMP post-intervention compared to the MICT group, further confirming the efficacy of high-intensity interval structures in improving anaerobic performance. The Cyclus2-derived FI—reflecting the rate of power decline from peak power to the subsequent minimum power during the 30-s Wingate test—decreased significantly following HIIT, indicating a slower power-decline rate (i.e., improved fatigue resistance and an enhanced ability to sustain high power output over short durations [55]. In contrast, the FI of the MICT group showed virtually no change, suggesting that traditional moderate-intensity continuous training offers limited stimulation of energy system regulation and lactate tolerance. These findings are consistent with previous research by Yamada et al. [56] and Chae et al. [57], which emphasized that only training programs with sufficient intensity and appropriately designed interval structures can induce significant physiological adaptations related to fatigue resistance. It should be noted that FI in the present study was obtained from the Cyclus2 software as a facility-specific, manufacturer-defined metric (W/s), which differs from the conventional percentage-based Wingate fatigue index (FI% = [(PP − Pmin)/ PP] × 100). Therefore, caution is warranted when directly comparing our FI (W/s) values with studies reporting FI% only.

From a performance perspective, the aerobic and anaerobic adaptations observed here should be interpreted in an integrated manner. In both HIIT groups, VO2max and Pmax improved alongside increases in RPP and RMP and reductions in FI, indicating not only greater peak power but also better ability to sustain high-intensity output. By contrast, MICT induced only modest aerobic changes and did not meaningfully alter Wingate-derived power or fatigue resistance. For speed skating, which requires a strong aerobic base together with repeated accelerations, cornering, and final sprints, this combined adaptation profile with slide board-based HIIT is likely more advantageous than the predominantly aerobic adaptations seen with MICT. Although on-ice performance (e.g., race times) was not directly measured, the use of a skating-specific slide-board modality suggests that these physiological improvements may translate into competitive benefits, particularly in middle-distance events.

In summary, slide board-based HIIT effectively improved both VO2max and Pmax, with the “4+1” structure of the HIIT2 protocol showing greater potential for enhancing aerobic power output within a short-term intervention. Both HIIT protocols also led to meaningful improvements in short-term power output and fatigue resistance, aligning well with the demands of speed skating events involving rapid starts, variable pacing, and final sprints. By contrast, the MICT protocol produced limited changes in these performance-related physiological indices over 4 weeks.

Beyond the context of speed skating, our findings are broadly consistent with HIIT interventions conducted in other athletic and clinical populations. Short-term HIIT has repeatedly been shown to elicit greater improvements in VO2max and aerobic performance than volume-matched or time-matched MICT in endurance athletes and team-sport players [18,50]. Similarly, Lasso-Quilindo et al. [51] reported that 4 weeks of HIIT enhanced VO2max, anaerobic power, and sport-specific performance in cyclists with cerebral palsy, despite their distinct neuromuscular and functional profiles. Taken together with these studies, the present results suggest that the superior effects of HIIT on both aerobic and anaerobic indices may represent a generalizable training principle that extends beyond cycling and running to skating-specific slide-board exercise. At the same time, the magnitude and pattern of adaptation are likely modulated by sport-specific biomechanical demands (e.g., crouched posture and lateral push-offs in speed skating), which underscores the importance of implementing HIIT using ecologically valid, discipline-specific modalities.

This study has several limitations that warrant further discussion. First, the small sample size (n = 9 per group) may have limited the statistical power to detect some interaction effects. Additionally, the absence of direct physiological mechanism indicators—such as blood lactate, muscle oxygen saturation, and enzyme activity—restricted deeper insights into energy system adaptations. Furthermore, the relatively short intervention period (4 weeks) did not allow for evaluation of the long-term maintenance or progression of training adaptations. In addition, total work duration and accumulated workload (time × intensity) were not equated across groups, which may have influenced the magnitude of between-group adaptations. Training intensity was also prescribed based on HR. While HR monitoring is practical and feasible in slide-board training environments, it is influenced by factors such as hydration, fatigue, and environmental conditions, and may not fully capture the intended metabolic stress. More precise methods, such as workload-, velocity-, or VO2-based prescriptions, or the use of individualized thresholds (e.g., lactate or ventilatory markers), are recommended for future studies [11]. Moreover, although slide-board skating is a practical skating-specific dryland modality, it cannot fully replicate on-ice mechanics (e.g., ice friction and glide characteristics), and we did not directly quantify the frictional properties of the slide-board surface; therefore, transfer to on-ice performance should be interpreted with caution. Lastly, the effects of the training interventions were not linked to actual performance outcomes (e.g., skating speed or sprint results), limiting the ability to assess the transfer of physiological gains to sport-specific performance.

Therefore, future research should extend in several directions. Expanding the sample size and adopting multicenter study designs could enhance the generalizability of findings. Incorporating cardiopulmonary, muscular, and metabolic mechanism assessments—such as near-infrared spectroscopy (NIRS), echocardiography, and electromyography—would provide more comprehensive insight into adaptation processes. Prolonging the intervention duration may help to clarify the long-term effects and functional transfer of different HIIT structures. Finally, integrating performance-based evaluations, such as on-ice sprint times or skating metrics, would improve the ecological validity and practical relevance of training outcomes.

In slide-board training for young speed skaters, both HIIT formats improved aerobic and anaerobic indices over 4 weeks, whereas MICT showed limited short-term change, suggesting that coaches may prioritize HIIT during short, sport-specific conditioning blocks while using MICT to maintain aerobic base. The 3/2 format (HIIT1), with longer relief, appears more conducive to increasing accumulated time at high VO2 and fatigue resistance, potentially aligning with middle- to long-distance demands, whereas the 4/1 format (HIIT2), with shorter relief, imposed higher glycolytic stress and yielded significant gains in Pmax, making it advantageous for sprint-oriented tasks emphasizing peak power. Nonetheless, these application suggestions should be considered tentative given the non-significant interactions for VO2max and Pmax and the small sample size; larger and longer trials linking physiology to on-ice performance (e.g., time trials) are warranted before prescriptive recommendations can be finalized.

## Conclusion

This study compared three slide board training modalities on aerobic and anaerobic performance in young speed skaters. Both HIIT protocols elicited significant improvements in VO2max and anaerobic power indices, whereas MICT produced no significant changes. Between-group differences were not statistically significant, although effect size estimates suggested greater short-term benefits with HIIT.

The HIIT2 protocol (4-minute work/1-minute recovery) yielded significant gains in Pmax, while HIIT1 (3-minute work/2-minute recovery) showed a smaller, non-significant trend toward improvement. These differences may reflect distinct physiological stresses imposed by the two formats, though such interpretations remain preliminary given the lack of significant interaction effects and the modest sample size.

Overall, the findings support slide board HIIT as a practical, sport-specific training method for young speed skaters, capable of enhancing both aerobic and anaerobic performance. The potential differential suitability of HIIT1 for endurance-oriented conditioning and HIIT2 for sprint-oriented development warrants confirmation in larger and longer-term trials, ideally including on-ice performance outcomes.

## Supporting information

S1 Dataset Individual pre- and post-training values of VO2max, Pmax, RPP, RMP, and FI for all participants by group (anonymized). (XLSX)
